# Ginkgolide B promotes osteoblast differentiation via activation of canonical Wnt signalling and alleviates osteoporosis through a bone anabolic way

**DOI:** 10.1111/jcmm.14503

**Published:** 2019-06-21

**Authors:** Bin Zhu, Feng Xue, Changqing Zhang, Guangyi Li

**Affiliations:** ^1^ Department of Orthopaedics Shanghai Jiao Tong University Affiliated Sixth People’s Hospital Shanghai China

**Keywords:** bone anabolic way, Ginkgolide B, osteoblast, osteoporosis, Wnt/β‐catenin signalling

## Abstract

Osteoporosis has become a worldwide problem as the population ages. Although many advances have been made in the treatment of osteoporosis in the past few years, the outcome are sometimes disturbing because of the adverse effects of these treatments. Further studies are still needed to identify novel alternate agents to improve the therapeutic effect. Ginkgolide B (GB), a derivative of *Ginkgo biloba* leaves, has numerous pharmacological effects, including anticancer and anti‐inflammation activities. However, the effect of GB on the regulation of osteoblast activity and bone formation effect has not yet been investigated. In this study, we showed the in vitro and in vivo effects of GB on osteoblast differentiation and bone formation. We found that GB promotes osteoblast differentiation of Bone Mesenchymal Stem Cells (BMSCs) and MC3T3‐E1 cells in vitro in a Wnt/β‐catenin‐dependent manner. In an in vivo study, we constructed a cranial defect model in rats and treated with GB. Histomorphometric and histological analyses confirmed that the usage of GB significantly promotes bone formation. Further study on ovariectomy (OVX) rats demonstrated that GB is capable of alleviating ovariectomy‐induced bone loss by enhancing osteoblast activity. Our findings indicate that GB is a potential therapeutic agent of osteoporosis through an anabolic way in bone.

## 1 INTRODUCTION

Osteoporosis is one of the most common bone disorders that affects people, and its incidence is dramatically increasing due to the population ageing.^1^ More than 20% of men over 50 years old and 40% of postmenopausal women worldwide are suffering from osteoporosis.^2^ Fractures, especially hip fractures and vertebral fractures, are common in osteoporotic patients due to decreased bone mass and strength.^3^ It has been reported that the mortality rate of hip fracture is nearly 20% during the first year of follow‐up observation.^4^, ^5^ Healthcare costs relating to osteoporosis and osteoporotic fractures are estimated to be nineteen billion per year.^6^, ^7^ Thus, osteoporosis carries a great financial burden on society.

Maintaining the balance between bone formation and resorption is essential for bone health.^8^ Osteoporosis occurs when bone resorption, which is mainly directed by osteoclasts, is faster than bone formation, mainly directed by osteoblasts.^9^ Current anti‐osteoporosis drugs mainly focus on decreasing bone resorption, while some undesirable effects hinder the therapeutic effect.^10^, ^11^, ^12^ For this reason, we try to focus on new therapeutic methods that promote bone formation, which is known as osteo‐anabolic therapy.^13^, ^14^, ^15^

The canonical Wnt/β‐catenin signalling pathway has been reported as a critical signalling pathway in bone development and homeostasis,^14^, ^16^ and it is one of the two main bone anabolic pathways that has been identified, along with the parathyroid hormone (PTH) signalling pathway.^15^, ^17^ Considering the critical role of Wnt//β‐catenin signalling in and its anabolic effect on bone homeostasis, we screened compounds for the treatment of osteoporosis by targeting the Wnt/β‐catenin signalling pathway. Ginkgolide B (GB) was isolated from *Ginkgo biloba* leaves*.* It has been demonstrated to exhibit anti‐tumour and anti‐inflammatory activities,^18^, ^19^, ^20^, ^21^ and it was closely related to Wnt signalling pathway.^22^ However, the exact role of GB in bone homeostasis and the underlying mechanism has not yet been fully elucidated. In this study, we attempted to investigate the effects of GB on bone formation and to explore its molecular mechanisms.

## 2 MATERIALS AND METHODS

### 2.1 Cell culture

Rat BMSCs were harvested from the marrow of healthy 4‐week‐old Sprague‐Dawley rats. A murine osteoblastic cell line (MC3T3‐E1) was purchased from the Chinese Academy of Science Cell Bank (Shanghai, China). Both of the above‐mentioned cells were seeded in T25 culture flasks (ThermoFisher, Shanghai, China) and maintained at 37℃ with 5% CO_2_ and cultured with a‐MEM (HyClone, Shanghai, China) supplemented with 1% penicillin/streptomycin (Gibco, Shanghai, China) and 10% foetal bovine serum (FBS, Gibco, Shanghai, China). All the procedures were approved by the Institutional Ethics Review Committee of Shanghai Sixth People's Hospital.

### 2.2 Cell toxicity and proliferation assay

The cell counting kit‐8 (CCK‐8, Dojindo, Kumamoto, Japan) assay was used to measure the viability of cells cultured in the presence of GB. Cells were seeded in 96‐well plates at a concentration of 5,000 cells per well and cultured with increasing concentrations of GB (0, 5, 10, 20 µM) for 7 days. Then 100 µL of a‐MEM and 10 µL of CCK‐8 were mixed and added to each well on days 1, 2, 3, 4 or 5. The absorbance of the wells at a wavelength of 450 nm was measured on a microplate reader (Mode 680, Bio‐Rad, Hercules, USA) after a 1‐hour incubation at 37℃.

### 2.3 Osteogenic differentiation

Osteogenic differentiation medium (Cyagen, Guangzhou, China) was used to induce osteogenic differentiation, and all the procedures were in line with the user manual. Briefly, MC3T3‐E1 cells and BMSCs were seeded in 24‐well plates and cultured in the abovementioned osteogenic differentiation medium containing one of several concentrations of GB (0, 5, 10, 20 µM). The osteogenic differentiation medium was replaced daily. Alkaline phosphatase (ALP) activity was measured after five days of culture with an alkaline phosphatase assay kit (Nanjing Jiancheng Bioengineering Institute, Nanjing, China), and the calcium deposits were measured by Alizarin red staining (Cyagen Biosciences, Guangzhou, China) after 2 weeks of culture.

### 2.4 RNA isolation and qRT‐PCR assays

Total RNA from MC3T3‐E1 cells and BMSCs were extracted using TRIzol Reagent (Invitrogen, Carlsbad, USA) in line with the user manual and quantified on a NanoDrop 2000 (Thermo, Waltham, USA). Complementary cDNA was synthesized through reverse transcription with the aid of a PrimeScriptRT Reagent kit (TaKaRa, Shiga, Japan). The qPCR assay was performed using buffers from Roche (Roche, Basel, Switzerland) on ABI HT7900 (Applied Biosystems, Australia). Expression levels were normalized to β‐actin. The primers were synthesized by BioTNT (BioTNA, Shanghai, China) and their sequences are listed below:

Rat: OPN_F: GCTGTCTTTGGCATCGTTT; OPN_R: CGTCCGTCTCTTGGATCTC; OCN_F: CTGCCCCTCCTGCTTAC; OCN_R: GGGTCCTCATGGTGTCTG; Axin2_F: CTCCCCAGATTCCCCTCT; Axin2_R: CAGGCAAACCAGAAGTCCA; Lef1_F: ACTGGCATCCCTCATCC; Lef1_R: CCTTTCTCTGTTCGTGCTG; Cnx43_F: ATGTGTTTCCCTCTTGCG; Cnx43_R: ATGAATGGATGGGCTAGGT; Osterix_F: AACTGGAGGGGAGTGGTG; Osterix_R: GGGCAGTCGCAGGTAGA;

Mouse: OPN_F: AGTCGATGTCCCCAACG; OPN_R: ACTCACCGCTCTTCATGTG; OCN_F: GACCTCACAGATGCCAAGC; OCN_R: CAAGGTAGCGCCGGAGT; Axin2_F: CGAGTGACGAATTTGCCT; Axin2_R: CGATCCTCTCCACTTTGC; Lef1_F: TATGAACAGCGACCCGTA; Lef1_R: CGGAGAAAAGTGCTCGTC; Cnx43_F: TCTGTCCCACCTTTGTGTC; Cnx43_R: CTTGCCTCCCTGATGCT; Osterix_F: AAACATCAGCGCACCCA; Osterix_R: GCAGGCGAAGTGGAAGAT.

### 2.5 Western blot analysis

Protein lysates from of MC3T3‐E1 cells and BMSCs were harvested using Cell Lysis Buffer supplemented with Protease Inhibitor (Boster Biological Technology, Wuhan, China), and the protein concentrations were quantified using a BCA Protein Assay Kit (Thermo, Waltham, USA). The lysates were separated on a 10% SDS‐PAGE gel (EpiZyme, Cambridge, MA) at 120V for 60 minutes and transferred to a polyvinylidene difluoride membrane (PVDF, Merck, Darmstadt, Germany) at 200 mA for 90 minutes. The membranes were then blocked using 5% milk in Tris‐buffered saline containing 0.1% Tween‐20 (TBST) for 90 minutes. Then, the membranes were incubated with primary antibodies at 4℃ for overnight followed by incubation with the corresponding secondary antibodies. The primary antibodies used in this experiment were listed as follow: Runx2 (Cell Signaling Technology, 1:3000); Type I collagen (Abcam, 1:5000); Osterix (Abcam, 1:2000); β‐catenin (Abcam, 1:5000); GSK‐3β (Signalway Antibody, 1:2000); GSK‐3β (Phospho‐Ser9) (Signalway Antibody, 1:1000) and GAPDH (Cell Signaling Technology, 1:4000). Goat Anti‐Rabbit IgG (H + L) (ProteinTech, 1:5000) served as the secondary antibody. After the membranes were treated with a chemiluminescence reagent (Thermo Fisher Scientific), Image Quant LAS 4000 (GE Healthcare) was used to detect the target bands.

### 2.6 Plasmid transfection and reporter gene activity assay

293T cells were seeded in 24‐well plates at a density of 1 × 10^4^ cells per well and transfected with Runx2, β‐galactosidase and OCN‐Luc plasmid using Lipo3000 (ThermoFisher, Waltham, USA) following the user manual. Super (8×) TOPFlash plasmid was a gift from Randall Moon. For TOPFlash assay, BMSCs and MC3T3‐E1 cells were seeded in 24‐well plates at a density of 1 × 10^4^ cells per well and transfected with the Super (8×) TOPFlash plasmid using Lipo3000. 24 hours after transfection, the culture medium was replaced with osteogenic differentiation medium containing one of several concentrations of GB. Forty‐eight hours later, the cells were lysed, and the luciferase activity was measured using a luciferase assay system (Promega, Wisconsin, USA).

### 2.7 Animal studies

All animal experimental procedures were performed on the basis of the guidelines of the National Institutes for Health Guidelines for the Care and Use of Laboratory Animals. The experiments were carried out with the approval of the Animal Research Ethics Committee of Shanghai Sixth People's Hospital.

For the cranial defect model, 6‐week‐old Sprague‐Dawley male rats were placed under anaesthesia, after which a 1.5 cm incision was made in the middle of the scalp. Upon exposure of the calvarium by blunt dissection, two defects with the diameter of 5 mm were created using an electric trephine. Totally 18 rats were randomly allocated into three groups: (a) Control group (n = 6): Matrigel was injected in the area of the cranial defect; (b) BMSC group (n = 6): BMSCs cultured in osteogenic differentiation medium for 7 days were added to Matrigel and injected into the area of cranial defect; and (c) BMSC + GB group (n = 6): BMSCs were collected and added to Matrigel after culturing for 7 days in osteogenic differentiation medium containing 20 µM GB, and injected into the area of the cranial defect. The incision was closed, and an intramuscular injection of antibiotics was given after surgery. To maintain the drug concentration, rats in the GB group received 20 mg/kg intraperitoneal injection of GB every other day, whereas the other groups received an equal volume of normal saline. Six weeks later, the rats were killed via anaesthetization, and the calvaria were obtained for examination.

For the postmenopausal osteoporosis model, eighteen 4‐week‐old female Sprague‐Dawley rats were randomly allocated into three groups: Sham group (n = 6), ovariectomy (OVX) group (n = 6) and OVX + GB group (n = 6). Rats were anaesthetized and the bilateral ovaries were excised in an ultraclean environment. For the OVX + GB group, the rats received 20 mg/kg intraperitoneal injection of GB every other day; for the Sham and OVX groups, all rats received an equal volume of normal saline. Six weeks later, the rats were killed by anaesthetization, and the femurs were obtained for examination.

### 2.8 Micro‐CT scanning

All samples obtained from the animal models were scanned at 9 µm resolution by a micro‐CT scanner (SkyScan 1176, Kontich, Belgium). Further analysis was performed using CTAn (Bruker MicroCT, Kontich, Belgium) to calculate the bone mineral density (BMD, mg/cm^3^), trabecular separation (Tb.Sp, mm), trabecular thickness (Tb.Th, mm), trabecular number (Tb,N, mm^−1^) and bone volume per tissue volume (BV/TV, %). Three‐dimensional images were reconstructed using CTVox software (Bruker MicroCT, Kontich, Belgium).

### 2.9 Histological observation

Half of the samples in each group were dehydrated in ascending concentrations of alcohol and embedded in poly‐methyl‐methacrylate (PMMA). The samples were sliced into 5 µm sections using a microtome (Leica, Heidelberg, Germany) for Van Gieson's staining and Goldner's trichrome staining. The stained sections were examined on a LEICA DM 4,000 microscope (Leica) and imaged. Osteoblasts appear as mononuclear cells with prominent nucleoli lining the bone surface.^23^, ^24^ Young osteoblasts appear robust and cuboidal, but flatten and eventually become lining cells when they complete the process of bone formation.^23^, ^25^ The number of osteoblasts in five sections per sample was counted by two people blinded to the treatments.

### 2.10 Statistical analysis

All results were obtained from three separate replicates and are shown as the means ± SD (standard deviation). The differences between groups were compared using an independent‐sample *t* test. The means of multiple groups were compared using one‐way analysis of variance (ANOVA). Data were presented with GraphPad Prism 5 software (GraphPad Software, La Jolla, USA). SPSS software (Version 16.0) (IBM Corporation, New York, USA) was used to perform the statistical analysis. *P* < 0.05 was considered statistically significant.

## 3 RESULTS

### 3.1 GB stimulates osteoblast differentiation in vitro

**Figure 1:**
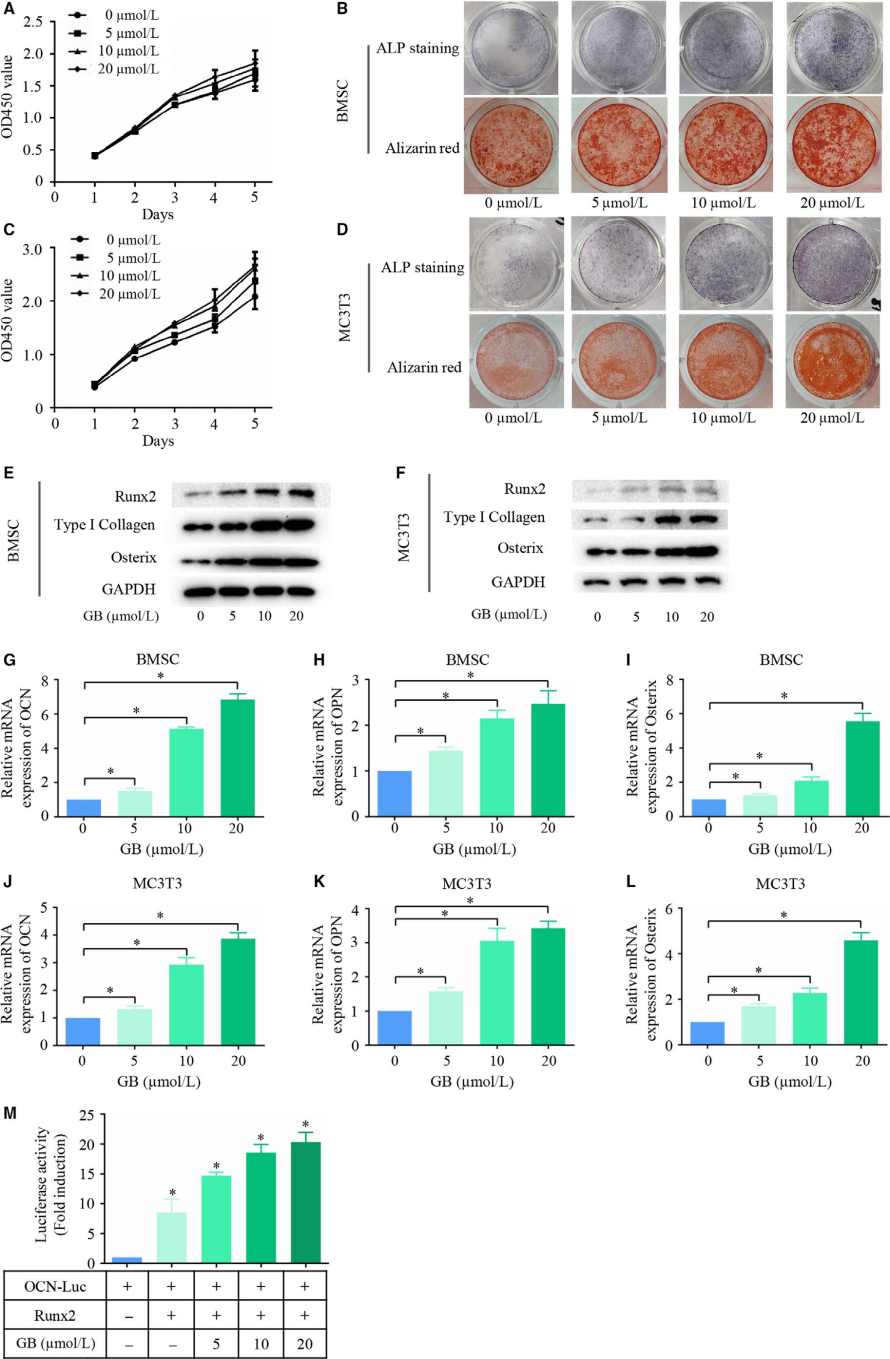
Ginkgolide B (GB) stimulates osteoblast differentiation in vitro. (A&C) The proliferation and viability of BMSCs and MC3T3‐E1 cells were assessed using CCK‐8 assay in the presence of up to 20 µM GB. (C&D) ALP staining and Alizarin red staining were performed separately on day 5 and day 14, respectively, to assess the effect of GB on osteoblast differentiation. (E&F) Runx2, COL1 and Osterix expression was measured by western blot assay after cells were cultured in osteogenic differentiation medium with one of several concentrations of GB for 5 d. (G‐L) OCN, OPN and Osterix expression was measured by qPCR after cells were cultured in osteogenic differentiation medium with one of several concentrations of GB for 5 d. The results were normalized to the expression of β‐actin. (M) Luciferase activity was assayed after treating with different concentrations of GB for 12 h. *P < 0.05

The CCK‐8 assay was used to assess the proliferation and viability of cells when GB was used at a dose up to 20 µM. GB showed no significant enhancing or inhibitory effect on the proliferation of BMSCs and MC3T3‐E1 cells. The influence of GB on osteoblast differentiation (Figure 1A,C) was further assessed. We first assessed the ALP activity, which is an important and frequently‐used indicator of initial osteoblast differentiation. ALP staining was performed 5 days after treatment with different concentrations of GB in osteogenic differentiation medium. We found that ALP activity was promoted in BMSCs by GB in a dose‐dependent manner (Figure 1B). These results showed that the initial osteoblast differentiation can be enhanced by GB. To further confirm this result, we also performed ALP staining in MC3T3‐E1 cells, and the results were similar (Figure 1D). Extracellular matrix (ECM) mineralization is the terminal step of osteoblast differentiation. To determine the effect of GB‐induced mineralization during osteogenesis, we performed Alizarin red staining (ARS) after 14 days of culture. The results showed that the number of plaques of calcified ECM gradually increased concomitant with the increase of GB concentrations in both BMSCs and MC3T3‐E1 cells (Figure 1B,D), which indicated that GB enhances the terminal phase of osteoblast differentiation by increasing mineralization. In addition, the expression of osteoblast differentiation marker genes, such as type I collagen (Col I), Runx2, Osterix, OCN, and OPN, were remarkably up‐regulated in both BMSCs and MC3T3‐E1 cells (Figure 1E‐L). To determine the effect of GB on the transcriptional activity of Runx2, 293T cells were cotransfected with Runx2 plasmid and OCN luciferase reporter and treated with varying concentrations of GB (0, 5, 10, 20 µM). As shown in Figure 1M, GB significantly enhanced Runx2 transcriptional activity at the OCN promoter in 293T cells. However, GB exhibits no promotion effect on adipogenesis or chondrogenesis of BMSCs (Figure S1).

### 3.2 The canonical Wnt signalling pathway is activated

**Figure 2:**
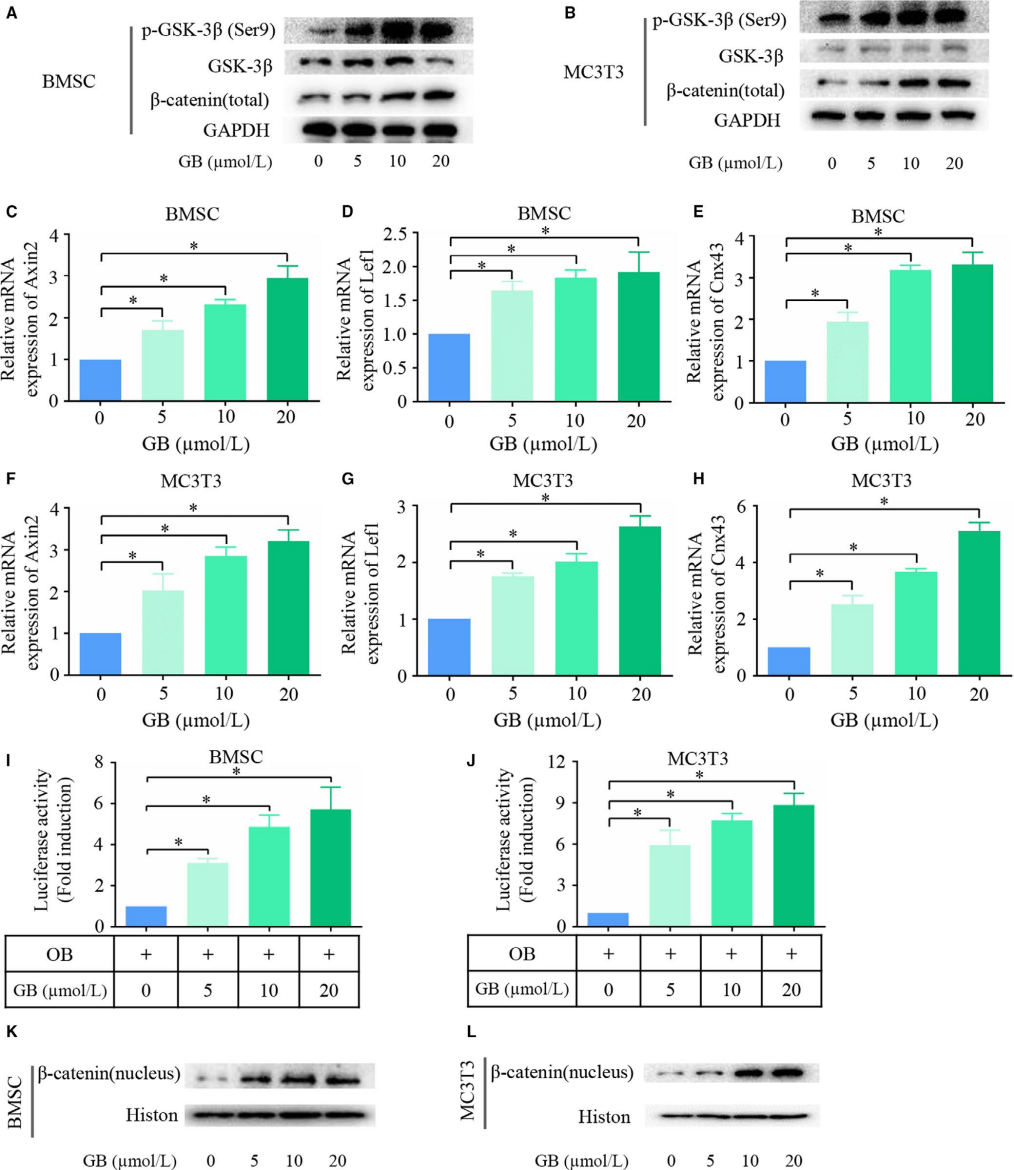
The canonical Wnt signalling pathway is activated. (A&B) GSK‐3β, p‐GSK‐3β (Ser‐9) and total β‐catenin expression levels were measured in BMSCs and MC3T3‐E1 cells. (C‐H) mRNA expression of Wnt/β‐catenin target genes (Axin2, Lef1, Cnx43) was measured by qPCR in both BMSCs and MC3T3 cells, and the results were normalized to the expression of β‐actin. (I&J) BMSCs and MC3T3‐E1 cells were co‐transfected with Super (8X) TOPFlash reporter and a Renilla control reporter. The cells were cultured in osteogenic differentiation medium with different concentrations of Ginkgolide B (GB) for 48 h. The cells were lysed and luciferase activity was measured. (K&L) Nuclear β‐catenin was measured by western blot in both BMSCs and MC3T3‐E1 cells. *P < 0.05

Canonical Wnt signalling plays a pivotal role in osteoblast differentiation. To confirm whether the Wnt signalling pathway was activated upon GB treatment, we first measured the expression of β‐catenin, a key factor in the Wnt/β‐catenin signalling pathway. As shown in Figure 2A,B, β‐catenin expression was significantly up‐regulated when the concentration of GB increased in both BMSCs and MC3T3‐E1 cells. Phosphorylation of GSK‐3β at Ser9 results in inactivation of its enzymatic activity, which is a crucial step for the activation of the canonical Wnt signalling pathway.^26^, ^27^ As illustrated in Figure 2A,B, the level of phosphorylation Ser9 on GSK‐3β was elevated by GB in a dose‐dependent manner, while the total GSK‐3β levels remained unchanged. Next, we examined the mRNA expression of Wnt/β‐catenin target genes in both BMSCs and MC3T3‐E1 cells. We found that the mRNA expression levels of Axin2, Lef1 and Cnx43 were up‐regulated when the concentration of GB was increased (Figure 2C‐H). TOPFlash reporter system was employed to further confirm the activation of the Wnt/β‐catenin pathway. As shown in Figure 2I,J, the luciferase activity was remarkably enhanced by GB. Taken together, Wnt/β‐catenin signalling pathway was activated in the process of GB‐mediated osteoblast differentiation.

To study the underlying mechanism by which GB regulates the canonical Wnt/β‐catenin signalling pathway, we examined the subcellular localization of β‐catenin. As shown in Figure 2K,L, the nuclear expression of β‐catenin in both BMSCs and MC3T3‐E1 cells was enhanced by GB. This effect was amplified when the concentration of GB increased, indicating that GB could induce the nuclear accumulation of β‐catenin.

### 3.3 GB promotes osteoblast differentiation in a canonical Wnt/β‐catenin‐dependent manner

**Figure 3:**
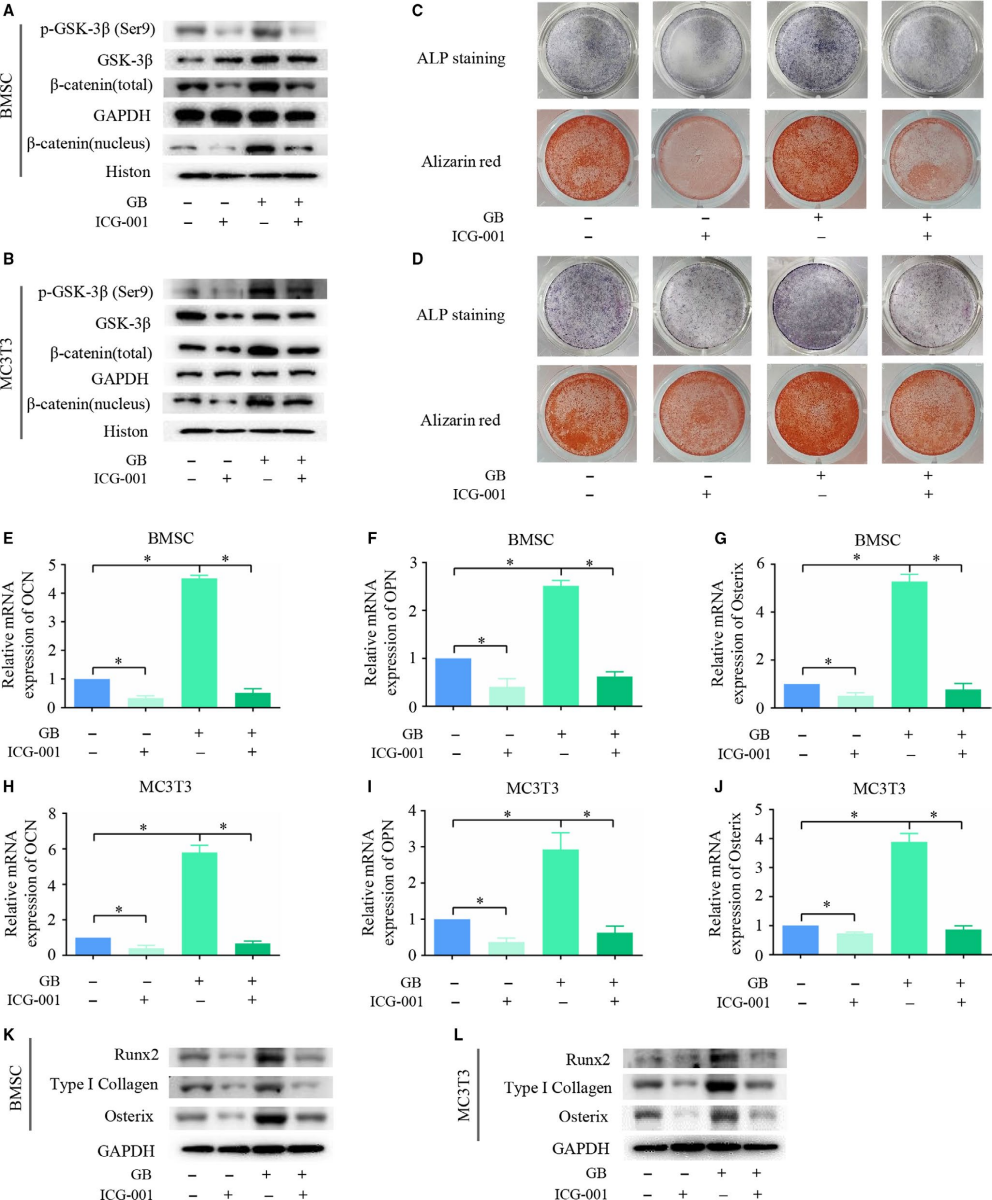
Ginkgolide B (GB) promotes osteoblast differentiation in a canonical Wnt/β‐catenin‐dependent manner. (A&B) GSK‐3β, p‐GSK‐3β (Ser‐9), total β‐catenin and β‐catenin (nucleus) expression was measured in BMSCs and MC3T3‐E1 cells treated with GB and/or ICG‐001. (C&D) ALP staining and Alizarin red staining were performed separately on day 5 and day 14, respectively, to assess the effect of GB on osteoblast differentiation in the presence of absence of ICG‐001 treatment. (E‐J) OCN, OPN and Osterix expression was measured by qPCR after the cells were cultured in osteogenic differentiation medium in the presence or absence of a mixture of GB and ICG‐001 for 5 d. (K&L) Runx2, COL1 and Osterix expression was measured by western blot assay after cells were cultured in osteogenic differentiation medium in the presence or absence of a mixture of GB and ICG‐001 for 5 d. The results were normalized to the expression of β‐actin. *P < 0.05

To study the potential role of the canonical Wnt/β‐catenin signalling pathway in GB‐mediated osteoblast differentiation, we blocked the pathway using ICG‐001, an antagonist of Wnt/β‐catenin/TCF‐mediated transcription. As shown in Figure 3A,B, ICG‐001 reduced the expression level of phosphorylated Ser9 on GSK‐3β in the presence or absence of GB in both BMSCs and MC3T3‐E1 cells. In addition, total β‐catenin and nuclear β‐catenin were both reduced, indicating that ICG‐001 significantly reduced the activation of the Wnt/β‐catenin signal pathway. Under this condition, ALP staining was performed 5 days after GB and/or ICG‐001 treatment. We found that the promotion of ALP activity by GB was weakened in both BMSCs and MC3T3‐E1 cells which were treated with ICG‐001 (Figure 3C,D). These results demonstrated that the initial osteoblast differentiation mediated by GB depended on activation of the Wnt/β‐catenin signalling pathway. Then, we assessed the ECM mineralization by ARS. As shown in Figure 3C,D, the promotion of mineralization by GB was also affected by ICG‐001 treatment in both BMSCs and MC3T3‐E1 cells. We also assessed the expression of osteoblast differentiation marker genes. We found that the GB‐mediated expression of Runx2, Col1, Osterix, OCN, OPN was significantly reduced by ICG‐001 (Figure 3E‐L). Taken together, these results indicated that GB promotes osteoblast differentiation in a Wnt/β‐catenin‐dependent manner.

### 3.4 GB promotes bone formation in vivo

**Figure 4:**
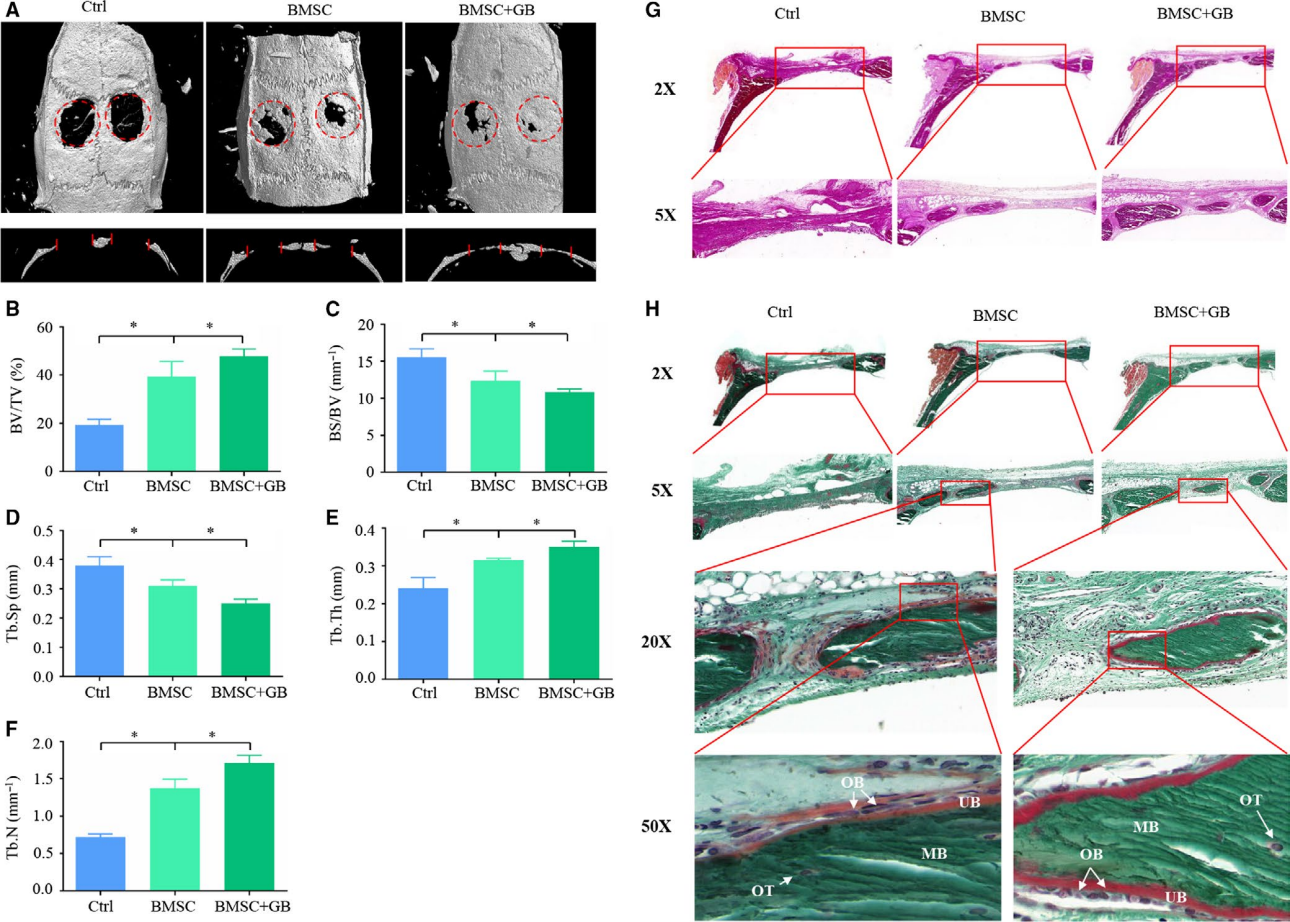
Ginkgolide B (GB) promotes bone formation in vivo. (A) 3D‐reconstruction and sagittal images of micro‐CT scans from different groups. (B‐F) Histomorphometric analysis of the defect area in different groups. (G) Van Gieson's staining was performed in each group. (H) Goldner's trichrome staining was performed in each group. *P < 0.05. (OB: Osteoblast; OT: Osteocyte; MB: Mineralized bone (green); UB: Unmineralized bone (red))

To analyse the effect of GB on osteoblast differentiation in vivo, we constructed a cranial defect model in rats. 3D‐reconstruction and sagittal micro‐CT images showed distinct bone formation in the defect area. We found that the area of newly formed bone tissue in the BMSC + GB group was greater than that in the Control or BMSC groups (Figure 4A). The histomorphometric analysis showed that GB treatment significantly increased the BV/TV, Tb.Th, Tb.N, and decreased the Tb.Sp, BS/BV (Figure 4B‐F). These results illustrated that GB promoted bone formation in vivo.

To further confirm these results, we performed a histological examination. Van Gieson's staining (Figure 4G) and Goldner's trichrome staining (Figure 4H) showed the newly formed bone tissue in each group. In addition, osteoblasts in the BMSC + GB group appeared cuboidal and secreted more osteoid, while the counterparts were flatten in the BMSC group, indicating that the GB administration significantly enhanced osteoblast activity.

### 3.5 GB alleviates OVX‐induced osteoporosis through a bone anabolic way

**Figure 5:**
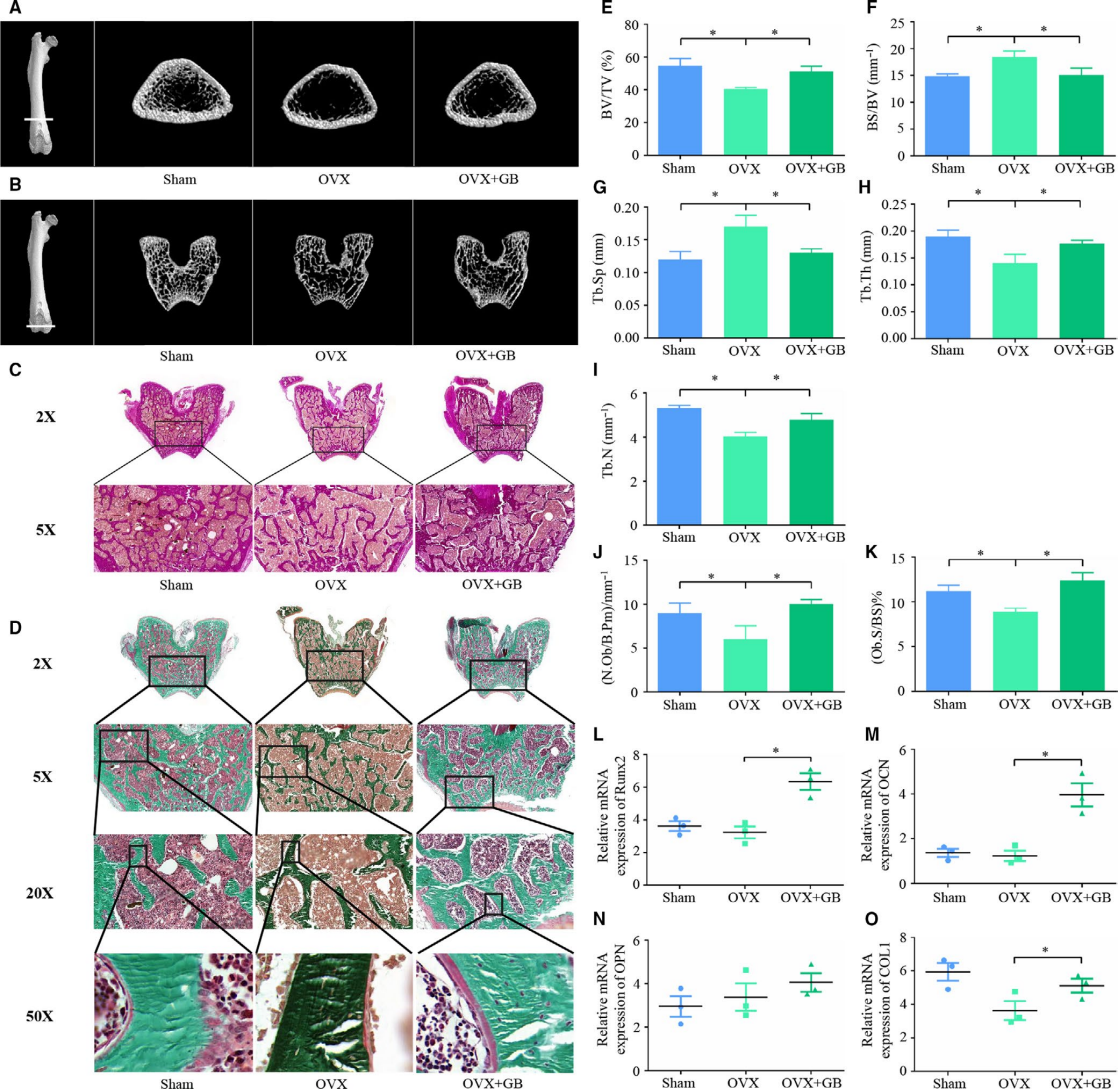
Ginkgolide B (GB) alleviates OVX‐induced osteoporosis through a bone anabolic way. (A&B) 3D reconstruction and transverse section images of micro‐CT scans of the different groups. (C) Van Gieson's staining was performed in each group. (D) Goldner's trichrome staining was performed in each group. (E‐I) Histomorphometric analysis of the defect area from different groups. (J&K) N.OB/B.Pm and Ob.S/BS were detected in sections subjects to Goldner's trichrome staining. (L‐O) Runx2, OCN, OPN and COL1 expression in femur samples from each group was measured by qPCR. The results were normalized to the expression of β‐actin. *P < 0.05

Because GB could promote osteoblast differentiation and bone formation in a Wnt dependent manner, we examined whether GB could alleviate osteoporosis through a bone anabolic pathway. To this end, OVX rats were treated with GB. 3D reconstruction and transverse micro‐CT images provided direct evidence that GB significantly increased the bone trabecula number in both the diaphysis and condyle of the femur (Figure 5A,B). Histomorphometric analysis also illustrated that the use of GB increased the BV/TV, Tb.Th, Tb.N, and decreased the Tb.Sp, BS/BV, demonstrating that the GB could alleviate OVX‐induced osteoporosis (Figure 5E‐I). Histologic examinations were performed to further confirm these results. Van Gieson's staining (Figure 5C) and Goldner's trichrome staining (Figure 5D) showed the bone tissue in each group, demonstrating that the use of GB alleviated OVX‐induced bone loss. In addition, Goldner's trichrome staining also showed that osteoblasts from the OVX + GB group appeared more robust and secreted more osteoid in comparison with the OVX group (Figure 5D). Furthermore, we detected the osteoblast number per bone perimeter (N.OB/B.Pm, mm^−1^) and osteoblast surface per bone surface (Ob.S/BS, %). As shown in Figure 5J,K, GB administration positively regulated osteoblast‐genesis in OVX‐induced osteoporosis. In addition, the expression of osteoblast differentiation marker genes was also measured in femurs obtained from each group. As shown in Figure 5L‐O, the Runx2, OCN, OPN and COL1 expression levels were increased, but the difference in COL1 expression was not statistically significant between treated and untreated groups.

## 4 DISCUSSION

Bone tissue is constantly engaged in metabolic activity to maintain a dynamic balance. The function of osteoblasts and osteoclasts in the maintenance and remodelling of bone is well‐organized and controlled under homeostatic conditions.^28^ Osteoporosis occurs when the process of bone resorption, which mainly directed by osteoclasts, is faster than the process of bone formation, which is mainly directed by osteoblasts.^28^, ^29^ The purpose of osteoporosis therapy is to improve the bone mass and strength to reduce osteoporosis related fractures. Therefore, bone resorption should be reduced and/or bone formation should be enhanced.^30^ Currently, most available anti‐osteoporosis therapies, such as anti‐RANKL antibodies and bisphosphonates, mainly focus on decreasing bone resorption. However, some undesirable effects are disturbing, such as osteonecrosis of the jaw,^31^, ^32^ atypical subtrochanteric femoral fractures^17^, ^33^ and orbital inflammation.^34^ We pay more attention to search for ways to increase bone formation, a process known as osteoanabolic therapy.^17^, ^35^ In this study, we showed that GB stimulates osteoblast differentiation and bone formation, exhibiting the potential to treat osteoporosis.

GB is a major constituent of the leaves of *Ginkgo biloba* and has been studied in many aspects. Hua et al^36^ demonstrated that GB might protect SH‐SY5Y cells against α‐Syn‐induced cell viability decrease. Jiang W and his colleagues found that GB could be proposed as an effective antiproliferative and apoptosis‐inducing agent with interesting translational application in ovarian cancers.^37^ Yao et al^38^ also found the anticancer effect of GB on prostate cancer. Besides, Zheng PD and his colleagues confirmed that GB could improve neurological function by promoting the proliferation and differentiation of neural stem cells in rats with cerebral ischemia/reperfusion injury.^39^ However, the effect of GB on osteoblast differentiation and bone homeostasis has not yet been studied.

To verify the effect of GB on osteoblast differentiation, we performed ALP staining and ARS, which reflect the initial and terminal phases of osteoblast differentiation, respectively.^40^ We also monitored the expression of osteoblast differentiation markers at the mRNA and protein level. To further determine the enhancing effect of GB on Runx2 transcriptional activity, we measured OCN‐luciferase reporter activity in 293T cells. Collectively, these studies demonstrated that GB could stimulate osteoblast differentiation in vitro. An in vivo experiment was conducted by using a cranial defect model in rats to further examine the efficacy of GB on bone formation. Through histomorphometric and histologic analyses, we found that GB significantly enhances the osteoblast activity and promotes bone formation.

The canonical Wnt/β‐catenin signalling pathway is widely studied and is critical in bone formation.^41^, ^42^, ^43^ In this study, the relationship between GB mediated osteoblast differentiation and Wnt/β‐catenin pathway was testified. We found that GB significantly enhanced the expression of key proteins in the Wnt/β‐catenin pathway and the expression of target genes were also promoted. The TOPFlash experiment further confirmed the activation of the Wnt/β‐catenin signalling pathway in GB‐mediated osteoblast differentiation.^44^, ^45^ Nuclear accumulation of β‐catenin is an important step for activation of target genes of the Wnt/β‐catenin signalling pathway.^46^, ^47^, ^48^ By examining the subcellular localization of β‐catenin, we confirmed that GB could induce its nuclear accumulation. We further blocked Wnt/β‐catenin signalling by using ICG‐001, a Wnt/β‐catenin pathway antagonist. ALP staining and ARS revealed that the promoting effect of GB on osteoblast differentiation was decreased. In addition, the expression of osteoblast differentiation marker genes was significantly reduced. Taken together, GB promotes osteoblast differentiation in a Wnt/β‐catenin‐dependent manner.

The Wnt/β‐catenin signalling pathway is one of the two main bone anabolic ways that have been identified.^14^, ^17^ For this reason, we wondered whether the use of GB can be used to treat osteoporosis through a bone anabolic pathway. Rats subjected to OVX‐induced osteoporosis were grouped and treated with or without GB. Six weeks later, we found GB administration markedly alleviated osteoporosis. The morphology of osteoblasts is an important indicator of osteoblast status. Young and energetic osteoblasts appear robust and cuboidal, and become flatten as their activity decreased, and eventually become lining cells.^25^, ^49^, ^50^ We noticed that osteoblasts in the OVX + GB group appeared more cuboidal and greater in number with more osteoid formation, when compared with the counterparts in the OVX group. Collectively, these results indicate that GB alleviates OVX‐induced osteoporosis through a bone anabolic method.

In conclusion, the study showed that GB promotes osteoblast differentiation and bone formation in a Wnt/β‐catenin‐dependent manner. Furthermore, GB notably alleviates OVX‐induced osteoporosis by enhancing both the number and activity of osteoblasts. This study demonstrates that GB might be a potential therapeutic agent against osteoporosis.

## CONFLICT OF INTEREST

All authors state that they have no conflicts of interest.

## AUTHOR CONTRIBUTIONS

Bin Zhu, Guangyi Li and Changqing Zhang designed the study; Bin Zhu and Feng Xue performed all the experiments with the help of Guangyi Li and Changqing Zhang; Bin Zhu and Guangyi Li analysed the results; Bin Zhu wrote the manuscript.
