# Supplementary figures and images for: Ginkgolide B promotes osteoblast differentiation via activation of canonical Wnt signalling and alleviates osteoporosis through a bone anabolic way
Source: J Cell Mol Med. 2019 Jun 21;23(8):5782–93. doi: 10.1111/jcmm.14503 (PMC6653448; doi:10.1111/jcmm.14503)

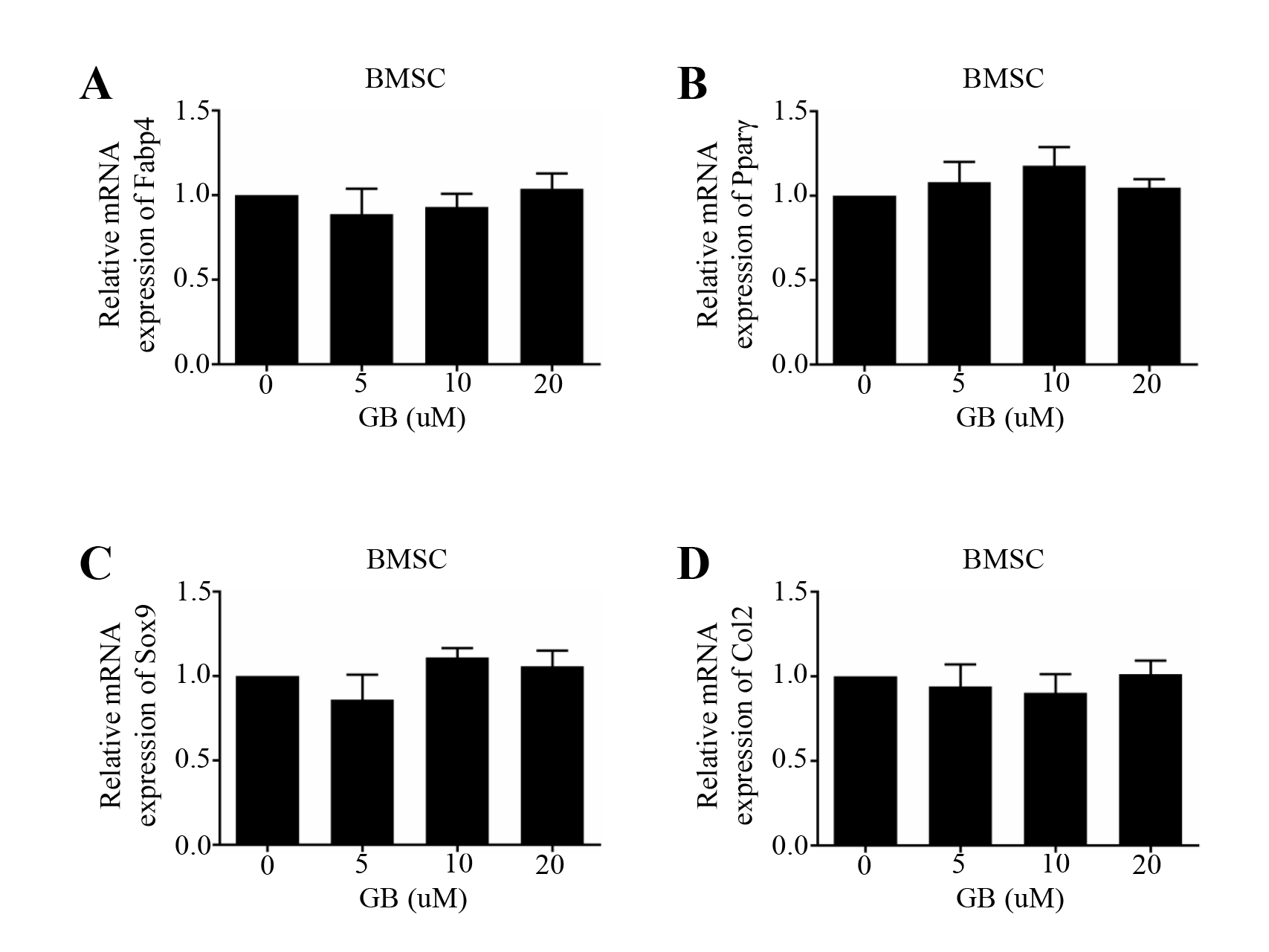

Supplement: Supplementary file 1 [file JCMM-23-5782-s001.tif]
